# Preventing fixation: Evidence of item-method directed forgetting protecting against mental impasses in creative problem-solving

**DOI:** 10.3758/s13423-024-02564-7

**Published:** 2024-09-16

**Authors:** Paula Gauselmann, Tobias Tempel

**Affiliations:** Ludwigsburg University of Education, Reuteallee 46, 71634 Ludwigsburg, Germany

**Keywords:** Creative problem-solving, Fixation, Item-method directed forgetting, Inhibition

## Abstract

Creative problem-solving can be impeded by mental impasses which are caused by fixation on associated but misguiding ideas. Recently, research has focused on the question of which processes might be involved in overcoming fixation and how it can be prevented from occurring altogether. In the present study, we investigated whether initially encoded fixation words can be forgotten by means of item-method directed forgetting, a procedure in which participants are presented with a series of items that are each followed by a remember cue or forget cue. Here, the series of items partly consisted of words that were misleadingly associated with compound remote associate problems that had to be solved later on. Two groups were compared: For one group, fixation words were followed by forget cues (fixation-F), whereas for the other group, they were followed by a remember cue (fixation-R). Results revealed three major findings: (1) The fixation-R group showed typical detrimental effects of fixation (i.e., impaired problem-solving performance), whereas there was no significant fixation effect in the fixation-F group, and (2) the magnitude of fixation was positively related to memory for fixation words. The present findings add to existing literature about how fixation negatively affects creative problem-solving and how it can be prevented by reducing the activation level of misleading thoughts.

**Supplementary Information:**

The online version contains supplementary material available at 10.3758/s13423-024-02564-7.

Creative problem-solving is an example of higher cognitive functioning that is characteristic of human intelligence. It typically involves engaging in associative thinking in order to generate innovative solutions to problems that cannot be solved by acquired routines. The fact that solutions are usually counterintuitive makes this process a challenging task—even more so when inappropriate associations hinder the thinking process, something which is known as mental fixation. This phenomenon can occur when misleading ideas are more closely linked in the associative network or when their associative nodes are more activated than those of potential solutions.

A well-established tool for investigating fixation in creative problem-solving is the Remote Associates Test (RAT; Mednick, 1962). The test includes multiple problems, each consisting of three cue words (e.g., ‘same’–‘tennis’–‘head’). The solver has to find an associative link (= solution) between these cues. This task is made especially difficult because each cue word is strongly linked to one or more misleading associations (e.g., ‘same’–*twin*, ‘tennis’–*ball*, ‘head’–*master*), whereas the solution is not intuitively accessible at first glance (e.g., for the previous example, the solution would be ‘match’). Several studies have demonstrated that exposure to potential fixation words before or during problem-solving attempts results in mental impasses, which significantly impede problem-solving performance (e.g., Beda & Smith, 2018; Kohn & Smith, 2009; Gauselmann, Frings, Schmidt et al., 2023, Gauselmann, Frings, & Tempel, 2024; Smith & Beda, 2020; Smith & Blankenship, 1991; Storm & Angello, 2010; Storm et al., 2011; Vul & Pashler, 2007).

Recent work has investigated how fixation can be overcome. In a broader sense, there are two general ways of doing so: by a shift of (mental) context or by targeted weakening of misleading associations (for a review on forgetting fixation theory and its practical implications, see Beda & Smith, 2022). Perhaps the simplest form of shifting one’s mental context is to put a problem aside for a while. This resting period, in which one can either stop engaging in problem-solving altogether (for example, clearing one’s head during a walk) or work on another task (which is both practical and economic), is referred to as *incubation* and typically results in better success during a second attempt of solving a problem (for a review, see Sio & Ormerod, 2009).

Whereas this approach implies that the problem-solving process has already been hindered by mental impasses, recent studies have shown that fixation can be avoided in the first place when the problem-solving context is different from the one in which exposure to fixation words took place (Beda & Smith, 2018; Gauselmann, Frings, & Tempel, 2024; Smith & Beda, 2020).

Besides these findings on the importance of contextual circumstances, inhibition of misleading ideas can play a crucial role in overcoming fixation during problem-solving. For example, May (1999) showed that the magnitude of fixation effects is affected by one’s circadian arousal, which is closely linked to one’s situational ability to inhibit interfering thoughts. Storm and Angello (2010) demonstrated that RAT problem-solving performance is positively correlated to individual differences in *retrieval-induced forgetting*, the phenomenon in which selective retrieval of a subset of previously encoded information renders the remaining, nonretrieved part of that information temporarily less available (Tempel et al., 2019). This type of temporary forgetting is thought to be triggered by an adaptive inhibitory mechanism acting to resolve interference during selective retrieval (e.g., Anderson, 2003; Anderson et al., 1994).

The fact that retrieval-induced forgetting and the ability to solve RAT problems are positively related to each other indicates that inhibitory processes seem to be involved in both. This assumption is further supported by a finding referred to as *problem-solving induced forgetting*. In a study by Storm et al. (2011), fixation was induced by letting participants memorize cue–response pairs, part of which consisted of cue words taken from subsequently presented RAT problems. After the problem-solving phase, participants received a memory test for all initially memorized cue–response pairs. Importantly, recall performance for problem-related response words was significantly worse than for problem-unrelated response words. This finding was interpreted to have resulted from inhibitory mechanisms that were activated during the problem-solving phase in order to suppress fixation on previously memorized problem-related response words.

Moreover, retrieval-induced forgetting can be used as a tool to prevent fixation from occurring in the first place. Inspired by a study by Gómez-Ariza and colleagues (2017), who showed that retrieval-induced forgetting of solution words can negatively affect RAT-problem-solving performance, a study from our lab showed that detrimental effects of induced fixation can be mitigated using a form of selective retrieval (Gauselmann, Frings, Schmidt, et al., 2023). F﻿﻿ixation﻿ was induced by letting participants initially memorize a list consisting partly of neutral and partly of fixation words. Before problem-solving, half of the participants selectively retrieved only the neutral words, which was supposed to induce forgetting of the nonretrieved fixation words. This was indeed the case, as indicated by enhanced performance for fixated problems compared with performance in a control group who showed typical detrimental effects of induced fixation. Similarly, a study by Angello and colleagues (2015) demonstrated that initially strengthened fixation words were less interfering during a word-fragment completion task after intentional item-by-item suppression using the Think/No-Think paradigm. In this paradigm, participants initially memorize a list of unrelated word pairs consisting of a target and a cue (e.g., ordeal–roach). This is followed by a Think/No-Think phase, in which they are subsequently presented with part of the target cues (the rest of the word pairs serve as baseline items) and instructed to either try to retrieve (think items) or to actively suppress and not think (no-think items) of the associated cue word. The typical finding is that, in subsequent direct and indirect memory tests, performance for no-think items is significantly worse than for both think- and baseline items, which has been seen as evidence for an inhibitory forgetting mechanism (Anderson & Green, 2001).

Another way for targeted weakening of memory traces are methods of directed forgetting. In the paradigm of list-method directed forgetting (LMDF; e.g., Sahakyan et al., 2013), participants study two word lists. The first list is either followed by a cue to be remembered or to be forgotten. Typically, subsequent memory for to-be-forgotten lists is significantly worse than for to-be-remembered lists. In a study by Koppel and Storm (2012), this memory impairment was used to reduce the activation level of initially memorized words that were misleadingly associated to word fragments that subsequently had to be completed. Blocking effects in the word-fragment completion task were significantly reduced for words that belonged to a list that had been cued to be forgotten. In another variant of directed forgetting, item-method directed forgetting (IMDF; e.g., Basden et al., 1993), participants are successively presented with a series of words that are each either followed by a remember cue or forget cue. While remember items have to be kept in memory for a subsequent recall test, forget items are to be forgotten. As in the LMDF paradigm, despite the instruction to forget certain items, participants usually are told to recall all of the initially presented items in a final memory test, and performance for forget items is significantly impaired compared with remember items. Two mechanisms in particular are assumed to contribute to this phenomenon: selective rehearsal of remember items (i.e., discontinued rehearsal of forget items; e.g., Basden et al., 1993) and inhibition of forget items after the cue was given (Anderson & Hanslmayr, 2014).

## The present study

The aim of the present study was to investigate whether IMDF can be used to prevent fixation in a similar manner as was demonstrated for LMDF, retrieval-induced forgetting and suppression-induced forgetting. This would add to both the understanding of fixation in creative problem-solving and the possibilities of reducing the activation level of misleading thoughts prior and/or during problem-solving attempts. We adopted the material and parts of the procedure from Gauselmann, Frings, Schmidt et al. (2023). Participants were presented with a series of words, each followed by a remember cue or a forget cue. In order to induce fixation, some of the words (fixation words) were misleadingly associated with some of the RAT problems that subsequently had to be solved. For one group, fixation words were forget items, whereas for the other group they were remember items. We expected to find typical detrimental effects of fixation during RAT problem-solving when fixation words were followed by a remember cue, but not when they were followed by a forget cue.

## Methods

### Participants

Seventy-two participants (32 women, 39 men, mean age = 24.2 years) took part in the experiment. Participants were recruited via Prolific, a platform for online recruitment. All participants were native German speakers and had a high school (or higher) degree. A required minimum sample size was calculated with G*Power (Version 2.1.9.7; Faul et al., 2007), assuming a medium-sized interaction effect and a desired power of 1 − ß = 0.80 (α = 0.05).

### Design

IMDF of fixation words was manipulated between participants. One group received cues to forget fixation words (fixation-F), and the other group received cues to remember fixation words (fixation-R). The dependent variable of interest was solution rates of target CRA problems (and nontarget CRA problems).

### Material

The experiment was designed using PsychoPy (Version 2020.1.3.), accessed via prolific.co, and run online on pavlovia.org via PsychoJS. Item material was taken from Gauselmann, Frings, Schmidt et al. (2023), comprising 12 CRA problems (four per trial—two target CRAs and two control CRAs) chosen from the 130 German CRA problems developed by Landmann et al. (2014) and three word lists with 16 items each.

A CRA problem is a slightly modified version of a RAT problem (Mednick, 1962), consisting of three seemingly unrelated cue words (nouns). Solving it requires finding a solution word that forms a compound with each of its cue words (e.g., problem: ‘palm’–‘shoe’–‘trunk’; solution: ‘tree’). Target and control CRA problems were matched regarding their average difficulty according to existing norming data (Landmann et al., 2014). Only moderately difficult problems were included in the study (i.e., only those solved within 60 s by 42.5% to 47.5% of the norming sample). A solution was rated as correct when it logically and lexically made sense—even if it was not correct according to its predefined solution (Landmann et al., 2014). This rating strategy has previously been validated by subjective evaluation of an independent sample (*n* = 30; for details, see Gauselmann, Frings, Schmidt et al., 2023).

Each list consisted of six misleading associations (= fixation words) and 10 neutral words (which were unrelated to both fixation words and CRA problems). As in the study by Gauselmann, Frings, Schmidt, and colleagues (2023), fixation words were created based on the target CRAs: Each target CRA was assigned three fixation words that were misleadingly associated with its three cue words, respectively. For example, the target CRA ‘Hütte’–‘Futter’–‘Steuer’ (English: ‘hut’–‘food’–‘tax’) with the solution word ‘Hund’ (English: ‘dog’) was assigned the following fixation words: ‘*Berg*’–‘Hütte’ (English: ‘*mountain*’–‘hut’), ‘Futter’–‘*Napf*’ (English: ‘food’–‘*bowl*’), ‘Steuer’–‘*Geld*’ (English: ‘tax’–‘*money*’). Importantly, control CRAs were neither associated with fixation words nor neutral words. Thus, fixation concerned only target CRAs, but not control CRAs.

Additionally (to being divided into neutral and fixation words), each word was chosen to be either a forget or remember item, which decided about whether the respective word would be followed by a forget or remember cue during the study phase (according to the typical IMDF procedure). Each list contained 10 remember items and six forget items. For the fixation-F group, the fixation words were forget items and the neutral words were remember items. For the fixation-R group, fixation words were remember items (alongside four neutral words), and six of the neutral words were forget items. This division was made a priori (i.e., it was not randomized).

### Procedure

**Fig. 1:**
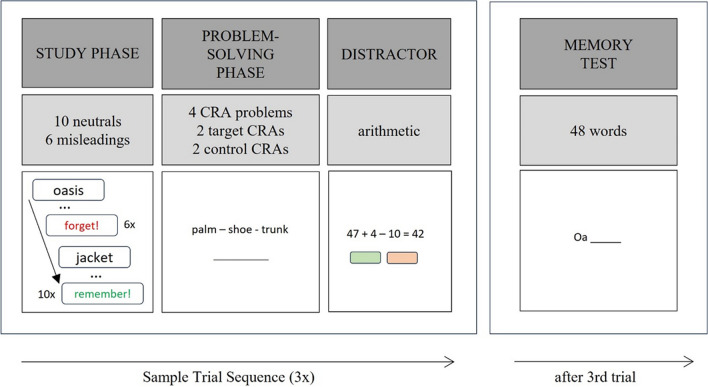
Sample trial sequence. *Note.* Each trial started with memorizing a word list, item by item. Each word was either followed by a forget cue (6×) or remember cue (10×). For the fixation-F group, fixation words were forget items. For the fixation-R group, fixation words were remember items. After having studied all 16 words, participants had to solve four CRA problems (2 target CRAs vs. 2 control CRAs). A trial finished with a distractor (mental arithmetic). At the end of the experiment, participants were tested on all 48 words, which surprisingly also included all forget items. (Color figure online)

The experiment was conducted online without guidance by an experimenter. The experiment link was accessed via prolific.co, which opened the experiment in full screen. Participants could navigate through the instructions at their own pace. To become familiar with the procedure, they practiced each task in a short practice trial, followed by a short feedback session to ensure comprehension. The experiment consisted of three trials with three phases each (study phase, problem-solving phase, arithmetic phase) and a final memory test (for an overview, see Fig. 1).

In the *study phase*, the words of one of the three lists (L1, L2, L3) were successively presented in randomized order in black font on a white screen for 1 s. Each word was preceded by three black dots that were presented for 2 s and followed by a blank screen for 3 s, after which the respective cue to either remember (‘*Merken!*’, English: ‘*Remember*’; green font) or forget (‘*Vergessen!*’, English: ‘*Forget*’; red font) the previous item was given. This was again followed by a blank screen for 3 s before the next word was displayed. In the beginning of the experiment, participants were told the importance of taking the cues seriously and to actively engage in the respective instruction (i.e., putting effort in either remembering or forgetting an item). They were also told that all remember items had to be kept in memory until the memory test at the end of the experiment.

In the *problem-solving phase*, participants had to solve four CRA problems—two target CRAs and two control CRAs, which were presented in random order. Each problem was presented for a total of 120 s. After typing a solution, participants were allowed to press ‘enter’, whereupon the next problem was displayed after a 500-ms blank screen.

The *arithmetic phase* at the end of a trial served as a distractor. Simple equations were subsequently presented for a total of 60 s. Participants had to evaluate whether the displayed solution was correct or not. Answers were given by clicking on the respective button (‘correct’ vs. ‘wrong’), whereupon the next equation was presented.

At the end of the experiment, participants were tested on all 48 words in random order—both the remember and (surprisingly) the forget items. Participants were told to recall all words, also forget items. Each word was cued with its first two letters. Solutions had to be typed in within 10 s, whereupon the next item was presented.

Half of the participants were randomly assigned to the fixation-F group, and the other half to the fixation-R group. The procedure and material were the same for all participants. The groups differed only in which items were remember items and which were forget items. Two trial sequences (trial 1: item set 1, trial 2: item set 2, trial 3: item set 3 vs. trial 1: item set 3, trial 2: item set 1, trial 3: item set 2) were counterbalanced between participants, resulting in four different versions of the experiment (two trial sequences per fixation-F group and fixation-R group respectively). After finishing the experiment and a first screening of the individual data by the experimenter, participants were compensated with £8,00 via prolific.co.

## Results

### Manipulation check

A paired- and a two-sample *t* test indicated that the crucial manipulation of forgetting fixation words was successful: the fixation-F group (but not the fixation-R group, *F* < 1) recalled significantly fewer fixation words (*M* = 15.71, *SEM* = 1.62) than neutral words (*M* = 45.01, *SEM* = 3.35), *t*(34) = −8.48, *p* < .001, *d* = −1.43, 95% CI [−1.90, −0.95] and also significantly fewer fixation words than the fixation-R group, *t*(69) = −5.70, *p* < .001, *d* = −1.35, 95% CI [−1.87, −0.83],﻿ fixation-R: *M* = 41.51, *SEM* = 4.18.

Data from one participant from the fixation-F group, who was an extreme outlier, were excluded from all analyses. Recall of forget items was not only four standard deviations above the mean but also higher than recall for remember items, indicating a lack of compliance with the directed-forgetting manipulation. Importantly, including the outlier in the analyses did not change the pattern of the results—that is, significant effects remain significant with or without outlier exclusion.

### Cued-recall performance (final memory test): Remember versus forget items

A 2 × 2 analysis of variance (ANOVA), with the between-participants factor *group* (fixation-F group, fixation-R group) and the within-participants factor *IMDF item type* (remember items, forget items) examined whether the two groups differed in their mean percentage of correctly recalled words during the final memory test, depending on the item type. The main effect of *IMDF item type*, *F*(1,69) = 89.21, *p* < .001, ηp^2^ = 0.56, reached significance. Remember items were recalled significantly more often than forget items (remember: *M* = 46.55, *SEM* = 2.61; forget: *M* = 19.82, *SEM* = 1.59). The main effect of *group* and the interaction of *group* and *IMDF item type* were not significant,* F* < 2.96 , *p* > .089.

### CRA solution rates (%)

**Fig. 2:**
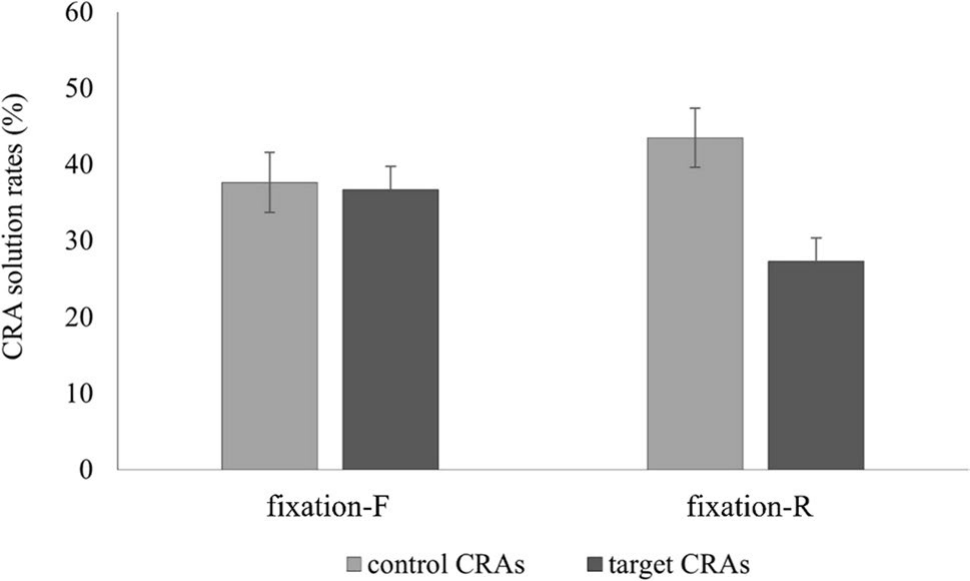
Mean CRA solution rates (%). *Note.* Mean CRA solution rates are displayed as a function of *group* (fixation-F vs. fixation-R) and *CRA type* (control vs. target). Error bars represent standard errors of the mean

A 2 × 2 ANOVA with the between-participants factor *group* (fixation-F group, fixation-R group) and the within-participants factor *CRA type* (target CRAs, control CRAs) examined whether the two groups differed in their mean percentage of correctly solved CRA problems, depending on the CRA type. The main effect of *group* was not significant, *F* < 1. However, the main effect of *CRA type*, *F*(1,69) = 17.33, *p* < .001, ηp^2^ = 0.20, as well as the interaction of *group* and *CRA type*, *F*(1,69) = 13.70, *p <* .001, ηp^2^ = 0.17, reached significance. Pairwise comparisons revealed that the fixation-R group solved significantly fewer target CRAs (*M* = 27.30, *SEM* = 3.03) than control CRAs (*M* = 43.50, *SEM* = 3.89), *t*(35) = −5.53, *p* < .001, *d* = −0.92, 95% CI [−1.28, −0.51], whereas no such difference was found for the fixation-F group (targets: *M* = 36.70, *SEM* = 3.07; controls: *M* = 37.60, *SEM* = 3.94), *t*(34) = −0.33, *p* = .744, *d* = −0.06, 95% CI [−0.39, 0.28] (see Fig. 2). Moreover, the fixation-R group solved significantly fewer target CRAs than the fixation-F group, *t*(69) = −2.17, *p* = .033, *d* = −0.52, 95% CI [−0.99, −0.04], whereas no significant difference was found for control CRAs, *t*(69) = 1.07, *p* = .291, *d* = 0.25, 95% CI [−0.22, 0.72].

### Exploratory analyses

**Fig. 3:**
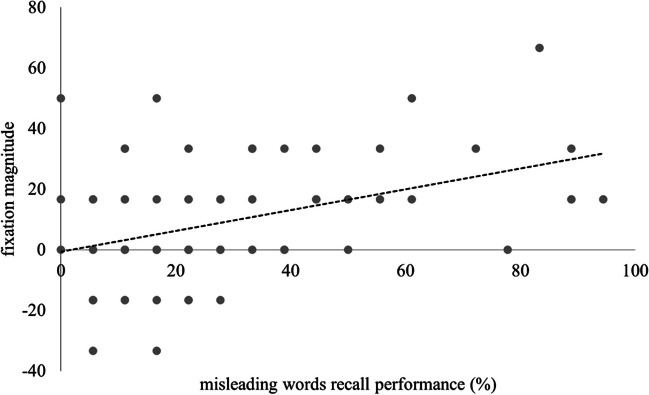
Scatterplot for recall performance of fixation words and fixation magnitude in problem-solving. *Note.* The scatterplot displays the positive relationship between recall performance for fixation words (%) and fixation magnitude (i.e., the difference between percentage of correctly solved control CRAs and correctly solved target CRAs)

For exploratory purposes, the correlation between recall for fixation words and fixation magnitude during problem-solving was analyzed. For this purpose, the difference between performance for control CRAs and performance for target CRAs was computed for each participant. Results revealed a positive correlation, *r* = .35, *p* = .003: The higher the percentage of recalled fixation words, the more fixation became apparent during problem-solving (i.e., the higher the difference between correctly solved control vs. target CRAs; see Fig. 3).

## Discussion

In the present study, we investigated whether fixation on misleading associations during problem-solving can be prevented by means of IMDF. Participants were presented with a series of words, some of which were supposed to induce fixation during subsequent problem-solving attempts (= fixation words). Each word was either followed by a remember cue or forget cue. For one group, fixation words were forget items, whereas for the other group, fixation words were remember items.

Results confirmed that fixation on misleading associations can be avoided by engaging in item-specific forgetting during encoding: While the fixation-R group showed typical detrimental effects of induced fixation, there was no significant fixation effect in the fixation-F group. They solved just as many CRA problems that were misleadingly associated to initially encoded fixation words (= target CRAs) as neutral CRA problems (= control CRAs).

This pattern corresponds to previous findings which have demonstrated that item-specific forgetting can render misleading associations less accessible during subsequent problem-solving and, thus, prevent detrimental impasses from occurring (retrieval-induced forgetting of fixation: Gauselmann, Frings, Schmidt et al., 2023; suppression-induced forgetting of fixation: Angello et al., 2015). Both retrieval-induced forgetting and suppression-induced forgetting have been attributed to retrieval inhibition by some researchers—that is, an inhibitory mechanism preventing currently irrelevant memory contents from interfering during retrieval of relevant memory contents (e.g., Anderson, 2003; Anderson et al., 1994; Anderson & Green, 2001), although a contribution of noninhibitory blocking processes has been suggested as well (Raaijmakers & Jakab, 2012).

The present findings do not allow us to decide whether inhibitory or noninhibitory mechanisms are more important in avoiding fixation in problem-solving. However, they demonstrate how a further targeted forgetting manipulation can reduce fixation, as retrieval-induced forgetting and suppression-induced forgetting have been shown to do. The discussion about underlying mechanisms of IMDF is controversial. While some studies support the notion that impeded memory for forget items is caused by discontinued rehearsal of forget items (e.g., Basden et al., 1993; Bjork, 1970; Tan et al., 2020), others speak in favor of an inhibitory mechanism preventing to-be-forgotten information from entering long-term memory (e.g., Fawcett & Taylor, 2008; Rizio & Dennis, 2013; Wylie et al., 2008). Some work also implies that both mechanisms contribute to IMDF (e.g., Anderson & Hanslmayr, 2014; Fellner et al., 2020).

Importantly, both assumptions imply that encoding depth for forget items should be substantially lower than for remember items, which in turn should result in (1) lower recall performance for forget items in both groups as well as (2) less interference by fixation words during problem-solving for the fixation-F group. While we are unable to answer whether they were caused by selective rehearsal or retrieval inhibition, both predictions were confirmed by our main findings. Future studies could compare the effects of different forgetting manipulations directly to determine which one reduces fixation most effectively. Perhaps the effect of IMDF will turn out to be the strongest because it affects storage of items early on, immediately after they were presented, whereas the effects of forgetting items due to later selective retrieval or suppression might be less reliable for weakening item representations. Recent meta analyses suggest that IMDF effects in general might be more robust than retrieval-induced forgetting or suppression-induced forgetting (Hall et al., 2021; Murayama et al., 2014; Stramaccia et al., 2021).

The importance of encoding for the magnitude of fixation effects had also been confirmed in a study by Beda and Smith (2018): Deeper encoding of fixation words resulted in higher subsequent problem-solving impairment. Exploratory results from the present study complement this finding: Correlational analyses revealed a positive relationship between recall performance for fixation words and fixation magnitude in problem-solving. The more fixation words participants remembered, the bigger the difference between the amount of correctly solved control CRAs and target CRAs. Thus, it can be assumed that the deeper the encoding of fixation words, the bigger their detrimental effect on subsequent problem-solving performance.

Besides their theoretical contribution, the present findings might have practical value because they imply that individual pieces of information can be prevented from interfering with creative problem-solving. Prompts for directed forgetting could enhance creativity. For example, when working together in a team to find a solution for a specific problem, the team leader might emphasize searching for relevant information in available documents or literature and also actively not retaining irrelevant information because it might include misleading associations.

Taken together, the present findings add evidence to what is currently known about how fixation impedes problem-solving outcomes. They extend previous findings on how fixation during the problem-solving process might be prevented.

## Supplementary Information

Below is the link to the electronic supplementary material.

Supplementary file1 (XLSX 12 KB)

## Data Availability

All data and materials have been made publicly available on the Open Science Framework (OSF) and can be accessed online (https://osf.io/cp85g/).

## Declarations

All procedures were performed per the ethical guidelines of the German Psychological Society (DGPs) and the APA Ethics code, in accordance with the ethical standards of the 1964 Helsinki Declaration and its later amendments or comparable ethical standards.
